# The evolution of intracranial aneurysm research from 2012 to 2021: Global productivity and publication trends

**DOI:** 10.3389/fneur.2022.953285

**Published:** 2022-09-28

**Authors:** Qian Zhang, Ling Weng, Jian Li

**Affiliations:** ^1^Department of Neurosurgery, Xiangya Hospital, Central South University, Changsha, China; ^2^Department of Neurology, Xiangya Hospital, Central South University, Changsha, China; ^3^National Clinical Research Center for Geriatric Disorders, Central South University, Changsha, China; ^4^Hydrocephalus Center, Xiangya Hospital, Central South University, Changsha, China

**Keywords:** intracranial aneurysm, bibliometric analysis, CiteSpace, VOSviewer, flow diverter, mechanism

## Abstract

**Background:**

This study aimed to analyze the global research trends and map the knowledge network of intracranial aneurysm (IA) research in the last 10 years.

**Methods:**

Publications related to IA from 2012 to 2021 were retrieved from the Web of Science core collection. Microsoft Excel 2010 and VOSviewer were used to characterize the largest contributors, including authors, journals, institutions, and countries. CiteSpace and VOSviewer were adopted to analyze the trends and knowledge network of IA.

**Results:**

A total of 5,406 publications related to IA from 2012 to 2021 were identified, increasing from 344 in 2012 to 762 in 2021. Siddiqui, AH from the USA contributed the most publications. Papers published in the journal World Neurosurgery ranked first in quantity, while Stroke ranked first for total citations and citations per publication. The top three prolific institutions were Capital Medical University, Mayo Clinic, and the University Department of Neurology Utrecht from 2012 to 2021. Moreover, the USA held the greatest share in the field, and China was almost on par with the USA due to its rapid growth. Specifically, the most frequently covered topics over the recent decade were subarachnoid hemorrhage, endovascular treatment (EVT), clipping, vascular disorders, flow diverter, stent, delayed cerebral ischemia, inflammation, and hemodynamics.

**Conclusion:**

The contribution made by different countries, institutions, journals, and authors for IA research over the past decade was demonstrated in the paper. The main topics include the choice of EVT or surgical clipping, particularly the application of flow diverter and associated complications, while themes such as the etiopathogenetic features of IA (e.g., inflammation and hemodynamics) deserve more attention.

## Introduction

Intracranial aneurysm (IA) is characterized by cerebral artery dilation, with a prevalence of 3–5% in the general population, and can be life-threatening if ruptured (1). With the advances in medical imaging and quality of life, patients increasingly are being screened for unruptured IA, which brings anxiety to patients and increases the health care burden on society (2). Recent research studies focused on the understanding and treatment of IA, which advanced the knowledge of IA; however, the exact pathophysiological mechanisms are largely unknown (3). The bibliometric analysis, which has gained popularity in recent years, is used to determine the contribution made by different authors, journals, institutions, and countries and discover the trend and hot spots in a particular field (4). The application of bibliometric analysis in the field of IA is fruitful. To be specific, Kiraz et al. (5) explored the papers related to IA from 1980 to 2020 analyzed by VOSviewer, with the research area limited to the category “neuroscience and neurology” and the search item “aneurysm” in the title, which excluded numerous IA-focused publications. Lu et al. (6) elaborated on the characteristics, content, and changes of the most prominent unruptured IA from the 100 most cited articles. Despite the ballooning biomedical scientific literature due to the advances in science and technology (7), the trend and knowledge map of the IA field almost remained untouched over the past decade. Therefore, this study adopted CiteSpace and VOSviewer, the widely accepted bibliometric analysis tools (8), to comprehensively explore the field of IA based on the retrieved publications from the Web of Science Core Collection (WoSCC) from 2012 to 2021, especially uncovering the following research questions (RQs) in the field of IA over the recent decade.

*RQ1*. What is the publication trend in the field of IA?

*RQ2*. What are the most influential articles and key authors, institutions, countries, and journals in this field?

*RQ3*. Who are the potential collaborators (authors, institutions, and countries/regions) in this field?

*RQ4*. What are the major themes and research frontiers in this field?

## Methods

### Search strategy

To avoid bias introduced by the database updates, a computerized search was performed in WoSCC (Thomson Reuters, New York, USA) from 1 January 2012 to 12 December 2021. The Science Citation Index-Expanded database contains indexed and peer-reviewed articles and basic information, including authors, affiliations, citations, and references. The literature search was carried out using the following items (“cerebral aneurysm^*^,” “intracranial aneurysm^*^,” or “cerebral aneurysm^*^”) in the title to filter out studies that focused on IA. Two investigators (Qian Zhang and Ling Weng) were responsible for the database search and filtering, while a senior neurosurgeon (Jian Li) was responsible for any discrepancies.

### Data extraction and bibliometric analysis

Bibliometric parameters, including title, keywords, journal, publication date, total citations, citations per publication, authors, institutions, and countries, were extracted, and were then imported into Microsoft Excel 2010 (Redmond, Washington, USA) for the analysis of contribution. VOSviewer (Leiden University, Leiden, the Netherlands) was adopted to visualize the mapping of coauthor-authorship, coauthor-institution, coauthor-country, coauthor-journal, and keywords co-occurrence. The node size in VOSviewer indicates the number of articles, while the width of links between the nodes indicates the cooperation strength (9). CiteSpace (Version 5.8. R1) was used to identify the keyword bursting and co-cited reference bursting to present the evolution of this domain (10).

The search only included documents published in English. Relative research interest (RRI) was defined as the number of publications about IA divided by the total number of publications per year in WoSCC (11).

## Results

### Overall characteristics

**Figure 1:**
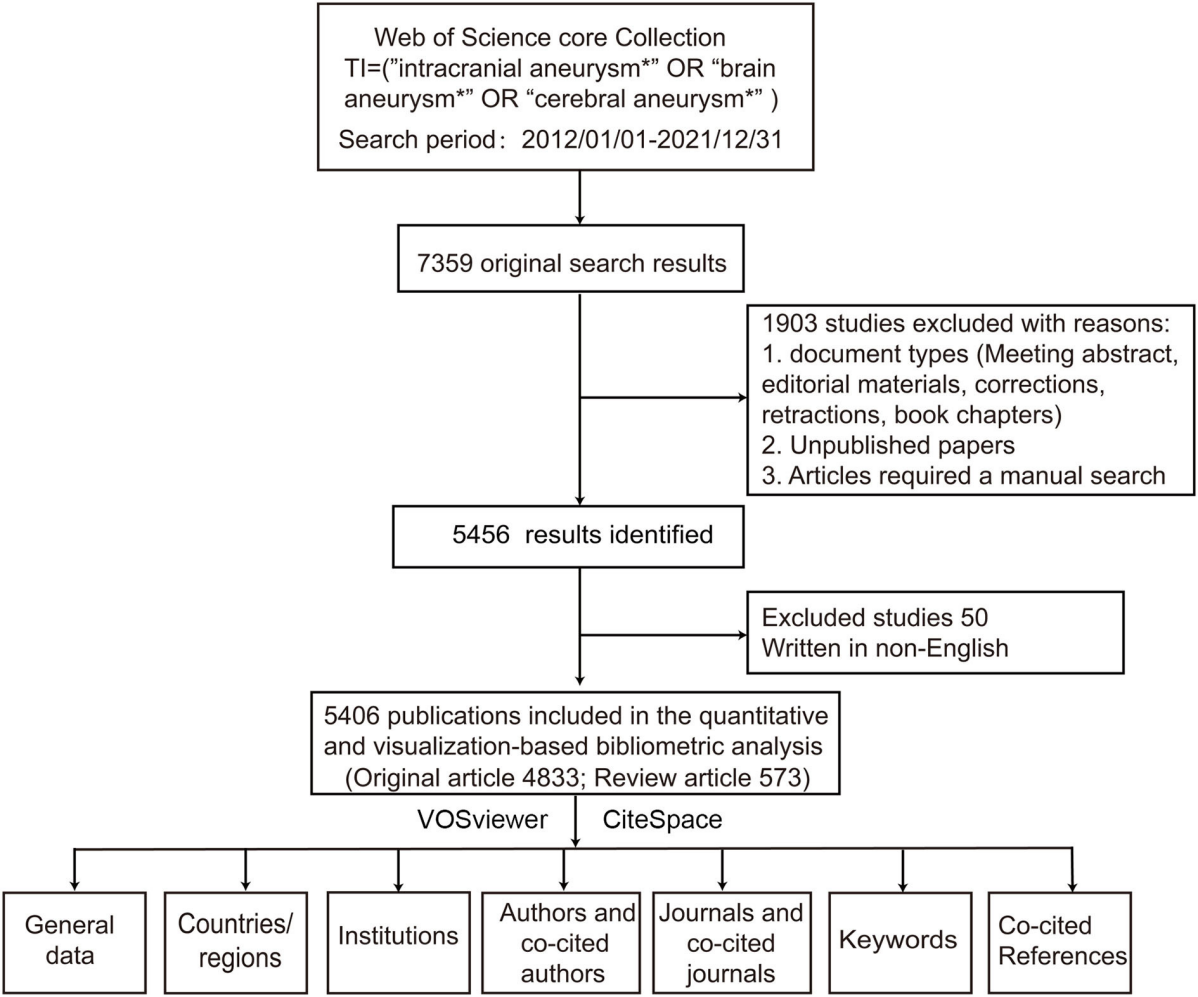
A flowchart of data screening and bibliometric analysis.

**Figure 2:**
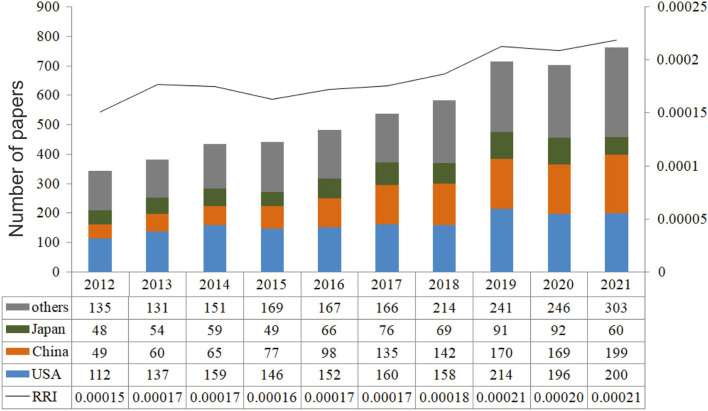
The number of publications of the top three prolific countries across time, and the time course of relative research interest of IA.

Figure 1 illustrates the 5,406 publications identified in the field of IA, most of which were original articles (*n* = 4,833), accounting for 89.4% of the total. The number of publications showed an upward trend, from 340 in 2012 to 762 in 2021, with a decline in 2020 (*n* = 703 publications). The total number of citations was 64,853, and the number of citations per publication was 12. A total of 99 countries/regions, 4,095 institutions, 19,436 authors, and 720 journals made their contributions to the field of IA. Figure 2 shows the countries that published the most papers (USA, China, and Japan) over the past 10 years.

### Countries/regions

**Figure 3:**
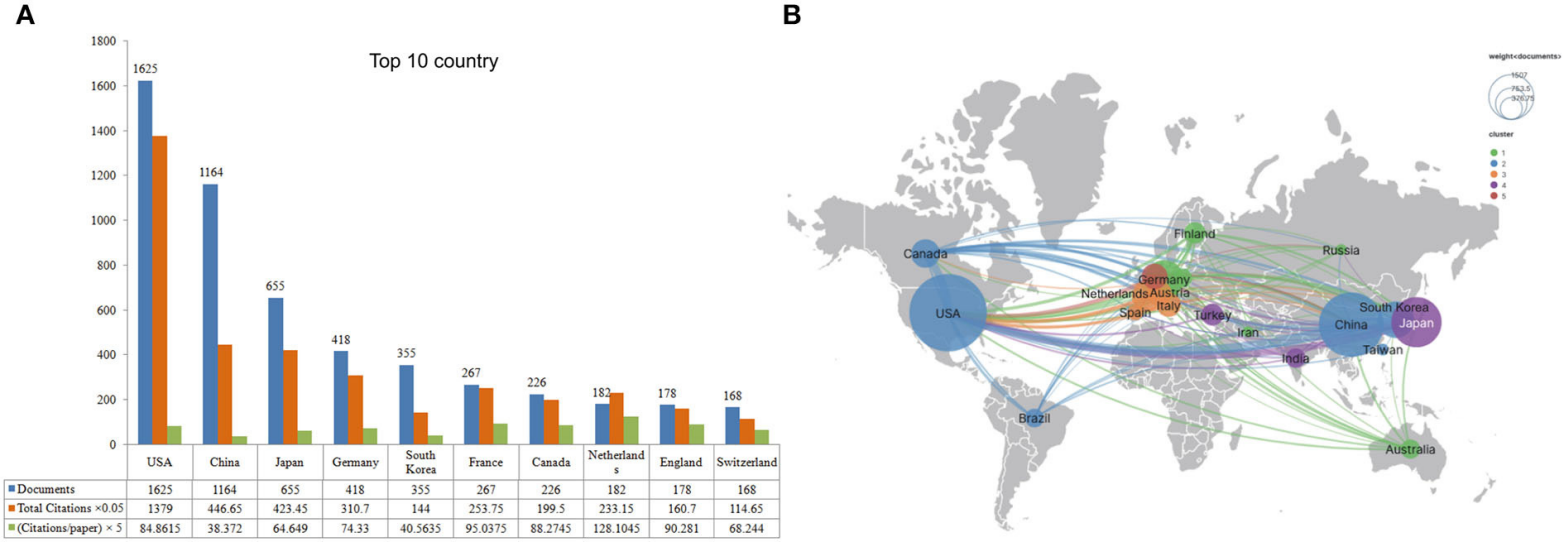
The top 10 prolific countries/regions and inter-national collaboration network of IA research from 2012 to 2021. **(A)** The number of publications, citations (×0.05), and citations per paper (×5). **(B)** The collaborative map of countries for IA. Node size indicates the number of articles produced. The width of links indicates the cooperation strength.

Figure 3A displays the top 10 productive countries/regions, with the USA taking the lead (1,625 publications; 27,580 citations). Next, VOSviewer was adopted to demonstrate the international collaborative map with the minimum publication set to 100. Finally, 14 countries met our criteria. The USA, China, Canada, Japan, and Germany presented as the center node. As seen in Figure 3B, the USA had the highest degree of cooperation with total link strength (TLS = 629). China (TLS = 98), Canada (TLS = 76), and Japan (TLS = 72) were the top three countries that had closer academic cooperation with the USA.

### Institutions

**Figure 4:**
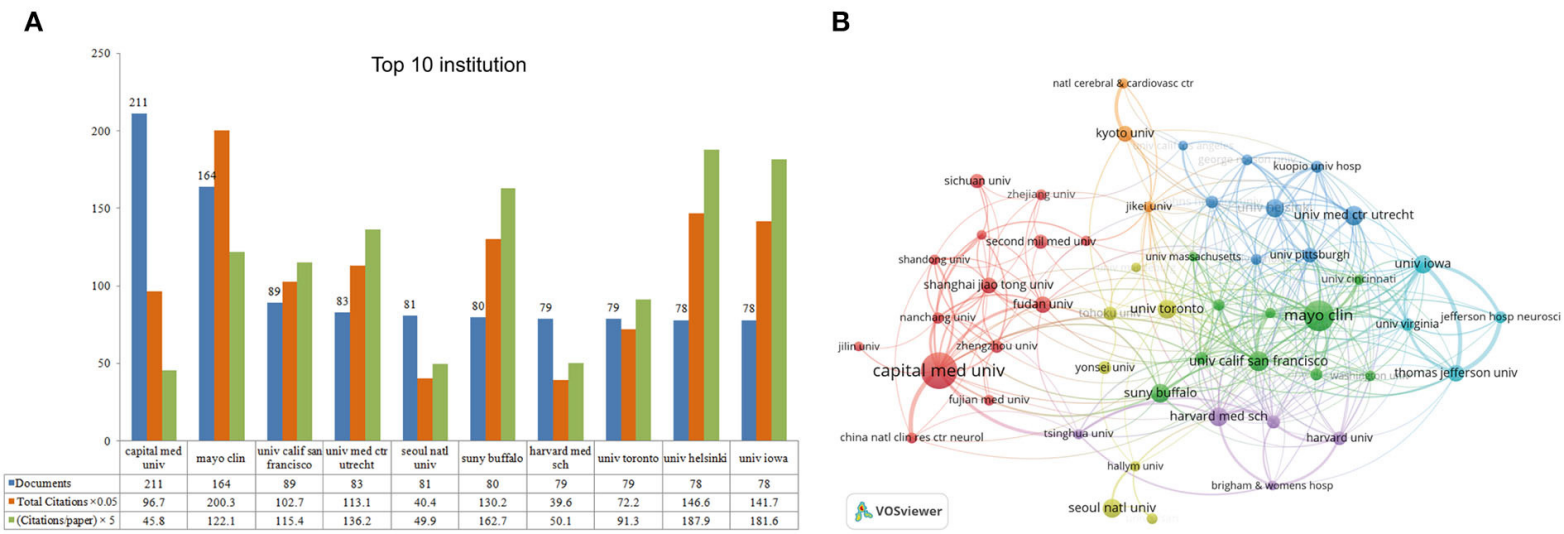
The top 10 most productive institutions and inter-institutional cooperation of IA research from 2012 to 2021. **(A)** The number of publications, citations (×0.05), and citations per paper (×5). **(B)** The cooperative map of institutions for IA visualized by VOSviewer. Node size indicates the number of articles produced. The width of links is positively associated with the cooperation strength.

Figure 4A shows the top 10 institutions, five of which were located in the USA. Capital Medical University ranked the first in the number of publications (China, *n* = 211), followed by Mayo Clinic (the USA, *n* = 164) and the University of California, San Francisco (the USA, *n* = 89). In terms of total citations and citations per publication, Mayo Clinic (*n* = 4,005; 24.4) ranked the first, followed by the University of Helsinki (*n* = 2,932; 37.6), and the University of Iowa (*n* = 2,833; 36.3). Figure 4B presents the VOSviewer results that visualized the cooperation between institutions, which show that 51 institutions with more than 30 published papers were selected for analysis, whose cooperation was distinctly geographically distributed. Mayo Clinic performed the strongest cooperation with 186 TLS, followed by Capital Medical University (TLS = 170) and the University of Iowa (TLS = 161).

### Journals and co-cited journals

The VOSviewer was used to perform a co-cited journal analysis to determine the most active and influential journals in the field of IA. A co-cited journal refers to a journal that has been cited by other journals in the same field. Table 1 describes the top 10 productive journals and co-cited journals, the former of which was led by World Neurosurgery with 606 publications (11.2%), Journal of Neurointerventional Surgery with 336 articles (6.2%), and American Journal of Neuroradiology with 255 articles (4.7%), while the latter by Stroke (16,447 co-citations), Neurosurgery (14,226 co-citations), and American Journal of Neuroradiology (14,069 co-citations).

**Table 1:** The top 10 prolific journals and co-cited journals on IA research between 2012 and 2021.

**Rank**	**Journal**	**Publications**	**Citations**	**Citations/paper**	IF*	**Co-cited journal**	**Co-citations**	IF*
1	World Neurosurg	606	4,289	7.0776	2.104	Stroke	16,447	7.914
2	J Neurointerv Surg	336	5,291	15.747	5.836	Am J Neuroradiol	14,266	3.825
3	Am J Neuroradiol	255	7,423	29.1098	3.825	Neurosurgery	14,069	4.654
4	J Neurosurg	216	3,748	17.3519	5.115	J Neurosurg	13,245	5.115
5	Neurosurgery	196	4,368	22.2857	4.654	J Neurointerv Surg	5,261	5.836
6	Interv Neuroradiol	180	1,030	5.7222	1.61	World Neurosurg	3,911	2.104
7	Stroke	142	6,430	45.2817	7.914	Acta Neurochir	3,045	2.216
8	Acta Neurochir	127	1,093	8.6063	2.216	Neuroradiology	3,033	2.804
9	J Clin Neuroscience	119	1,041	8.7479	1.961	Lancet	2,913	79.324
10	Neuroradiology	96	1,801	18.7604	2.804	Radiology	2,364	11.104

IF*:impact factor (2020).

### Authors and co-authors

**Figure 5:**
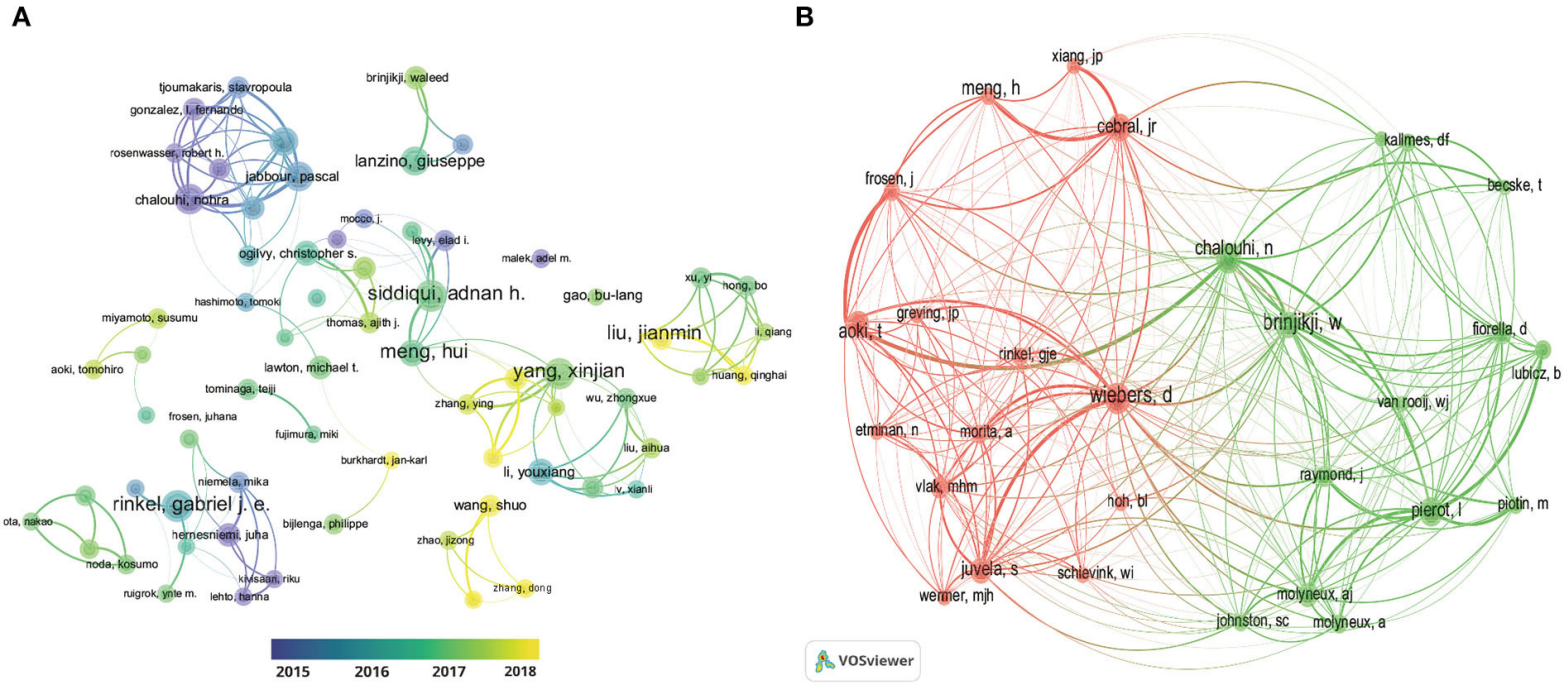
Author cooperation network and co-cited author network on IA studies from 2012 to 2021. **(A)** Collaborative map of prolific authors for IA studies from 2012 to 2021. **(B)** Co-cited authors of IA studies. Node size indicates the number of articles produced. The distance between any two nodes positively associates with the cooperation strength.

Table 2 lists the top 10 most productive authors and most co-cited authors (influential research teams and potential research partners), most of whom are from the USA. Siddiqui AH (*n* = 67 publications) from Suny Buffalo Department of Neurosurgery (USA) and Yang Xinjian (*n* = 61 publications) from Capital Medical university (China) and Gabriel Rinkel JE (*n* = 59 publications) from University Department of Neurology Utrecht (Netherlands), were the top three prolific authors between 2012 and 2021. A co-cited author was defined as an author who was co-cited in publications, and co-citation directly reflects the extent of an author's contribution. Wiebers D, Chalouhi N, and Laurent P were the three most co-cited authors in this field. Figure 5A depicts the author cooperative map obtained from VOSviewer, where Hernesniemi J and Chalouhi N were colored dark blue (the average year of publication in 2014–2016), indicating that they were active in the early phase, while Xinjian Yang, Jianmin Liu, Aihua Liu, and Shuo Wang were colored yellow-green or yellow (the average year of publication in 2017–2018), suggesting their active role in the late phase in the past 10 years. Figure 5B displays the top co-cited authors who received more than 300 co-citations.

**Table 2:** Top 10 prolific authors and co-cited authors on IA research from 2012–2021.

**Rank**	**Author**	**Publications**	**Citations**	**Citations/ paper**	**Country**	**Co-cited author**	**Co-citations**	**Country**
1	Siddiqui, Adnan H.	67	1,335	19.9	USA	Wiebers, D	1,210	USA
2	Yang, Xinjian	61	7,93	13.0	China	Chalouhi, N	1,201	USA
3	Rinkel, Gabriel J. E.	59	1,339	22.7	The Netherlands	Pierot, L	1145	France
4	Jabbour, Pascal	55	1,774	32.3	USA	Cebral, Jr	1,028	USA
5	Chalouhi, Nohra	54	2,513	46.5	USA	Brinjikji, W	929	USA
6	Lanzino, Giuseppe	53	1,353	25.5	USA	Aoki, T	850	Japan
7	Starke, Robert M.	52	1,768	34.0	USA	Juvela, S	850	Finland
8	Li, Youxiang	47	661	14.1	China	Molyneux, Aj	735	England
9	Meng, Hui	46	961	20.9	USA	Vlak, Mhm	682	The Netherlands
10	Ogilvy, Christopher S.	41	876	21.4	USA	Raymond, J	644	Canada

### Keywords

**Figure 6:**
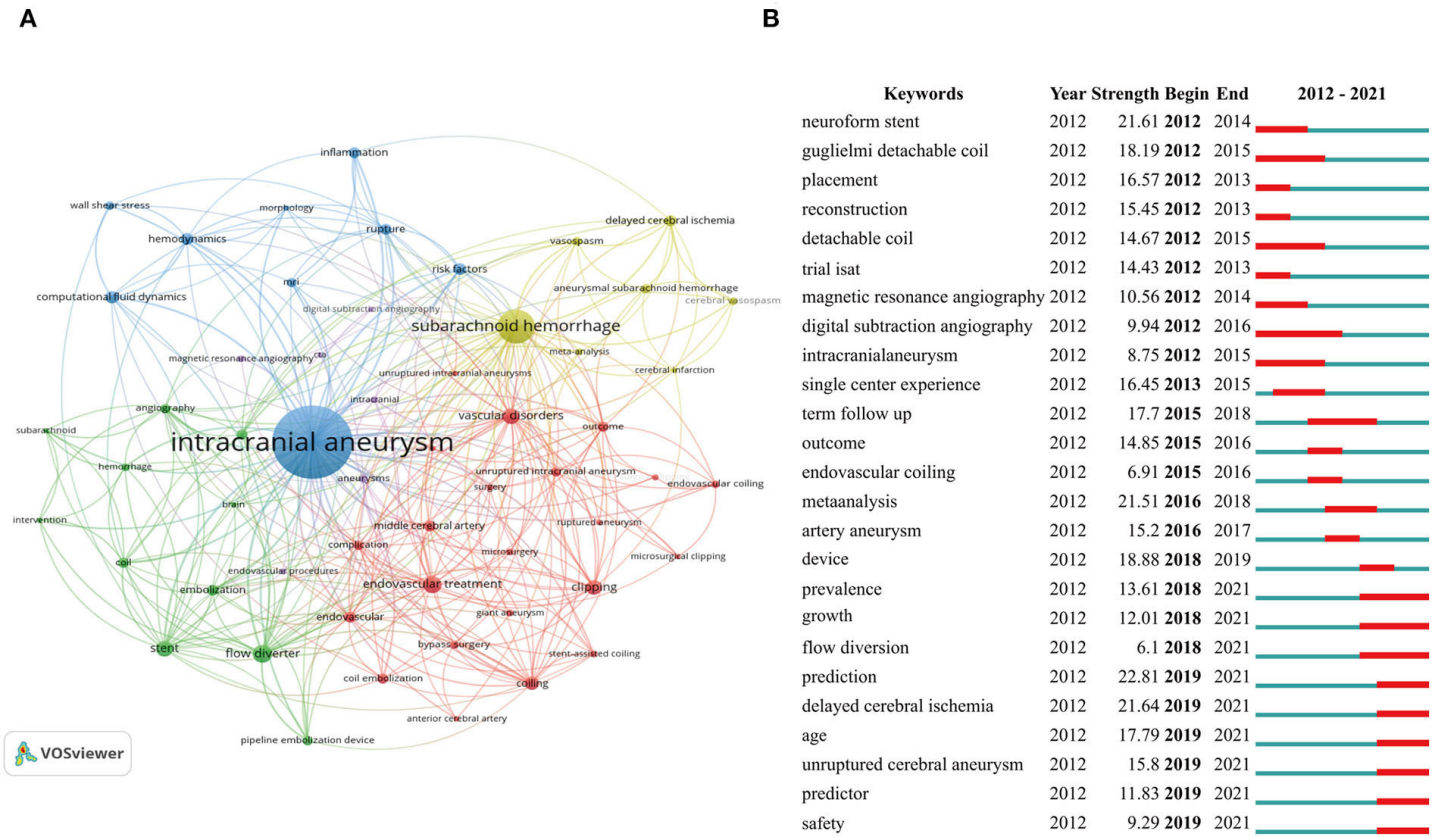
Analysis of keywords related to IA studies from 2012 to 2021. **(A)** The keyword co-occurrence network of IA studies in the recent 10 years. The keywords clustered into five groups according to their color. Large nodes represent keywords with high frequency; **(B)** The top 25 keywords with the strongest citation bursts on IA studies from 2012 to 2021 were displayed by CiteSpace. The red segment on the blue line denotes the burst duration.

The keyword co-occurrence network was adopted to describe the knowledge map and frontier topics in a field. Similar keywords were merged using a thesaurus (Supplementary Table S1), for example, brain aneurysm and cerebral aneurysm were replaced by IA. In total, 55 keywords appearing at least 40 times were collected and grouped into the following clusters, which were named based on their respective characteristics. As seen in Figure 6A, cluster# 1 (red and green) represented the research on the clinical treatment of IA, with “endovascular treatment, clipping, coiling, stent, and flow diverter” serving as the most frequent keywords; cluster# 2 (blue) indicated research on the mechanisms of IA, with the most frequent keywords including “hemodynamics, computational fluid dynamics, inflammation, and rupture;” cluster# 3 (yellow) represented research on complications of IA, with most frequent keywords including “subarachnoid hemorrhage (SAH), delayed cerebral ischemia, and cerebral vasospasm”; cluster# 4 (purple) indicated imaging research in IA, whose most frequent keywords covered “magnetic resonance angiography, CTA, and DSA.” In addition, CiteSpace was employed to visualize the distribution of keywords in different periods (Figure 6B), which showed that the topics related to “neuroform stent, detachable coil, and reconstruction” were focused on the early stage of the recent decade, while themes like “flow diversion, prediction, delayed cerebral ischemia, age, growth, predictor, safety, and unruptured cerebral aneurysm” acquired more attention between 2018 and 2021.

### Top cited articles and co-cited references

**Figure 7:**
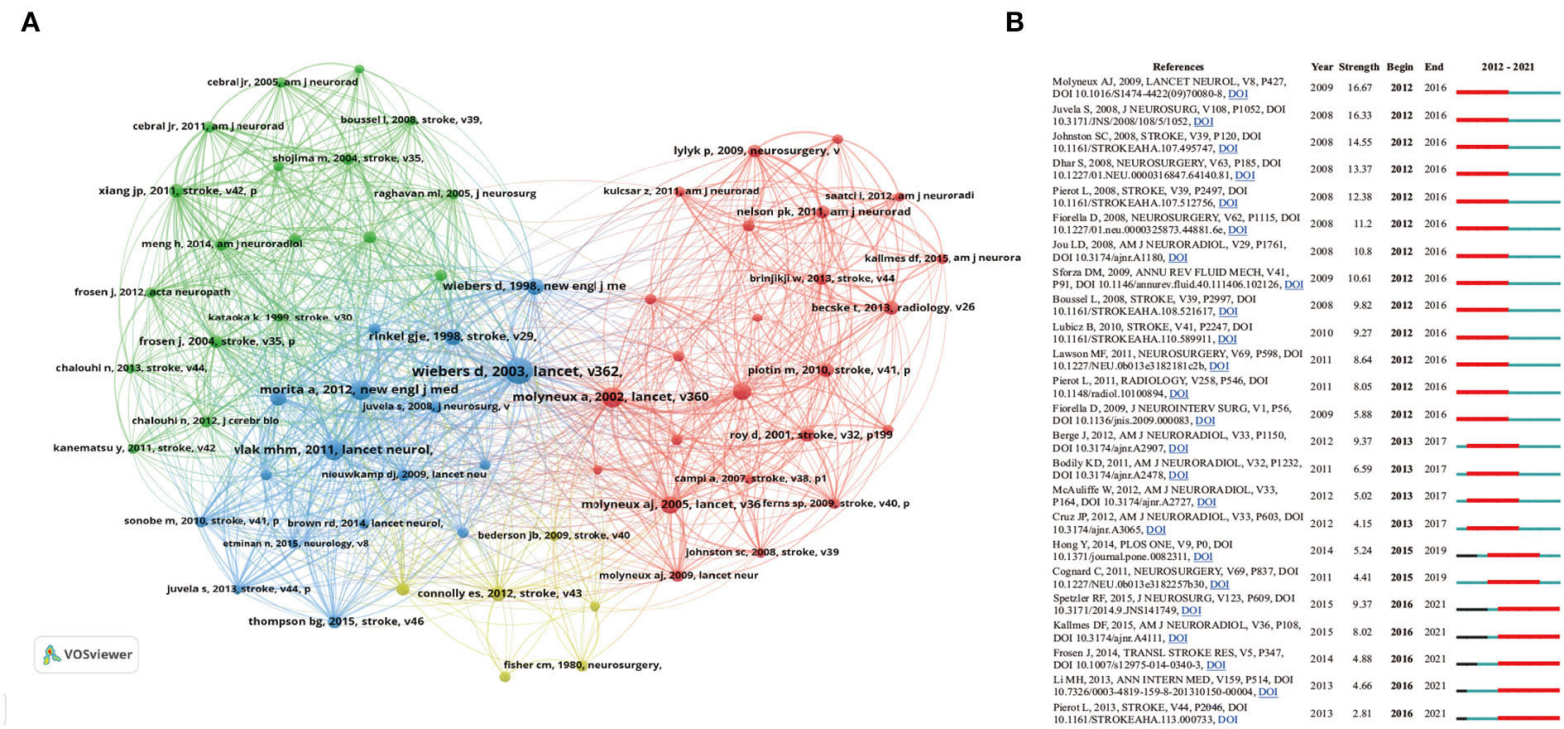
**(A)** The co-citation references network on IA studies from 2012 to 2021. **(B)** The top 24 references with the strongest citation bursts on IA studies from 2012 to 2021 were displayed by CiteSpace. The blue line represents the time from its first appearance to 2021, and the red line represents the burst time.

Table 3 lists the top 10 cited articles, involving six clinical research, two guidelines, and two reviews regarding the pathology and biology of IA. Morita et al. (12) published the most cited article in 2012 in the New England Journal of Medicine with 765 total citations, entitled *The Natural Course of Unruptured Cerebral Aneurysms in a Japanese Cohort*. This study conducted 11,660 aneurysm-years of follow-up on 5,720 patients with newly identified unruptured IA in Japan. It also found that the annual rupture rate was 0.95% in unruptured IA, and the rupture rate of unruptured IA is positively related to aneurysm size, daughter sac, and posterior circulation. Co-cited references were references co-cited by other literature studies (13). We identified 55,431 co-cited references from 5,406 publications, among which 65 references co-cited over 100 times were utilized to form a co-citation network. According to Figure 7A, the most-cited article (*n* = 826 co-citations) was entitled *Unruptured Intracranial Aneurysms: Natural History, Clinical Outcome, and Risks of Surgical and Endovascular Treatment* and was published by Wiebers et al. (1) in Lancet. Given that the reference burst in CiteSpace reflected researchers' interest in a certain article during a specific time period (10), we identified 24 references that had the strongest citation bursts, with the burst duration preset to be 5 years (Figure 7B). Of which, Molyneux AJ, 2009, LANCET NEUROL, V8, P427 (14) had the highest burst strength (*n* = 16.67 citation bursts), and articles with citation bursts ending in 2021 were as follows: Spetzler RF, 2015, J NEUROSURG, V123, P609 (15), Kallmes DF, 2015, AM J NEURORADIOL, V36, P108 (16), Frosen J, 2014, TRANSL STROKE RES, V5, P347 (17), Li MH, 2013, ANN INTERN MED, V159, P514 (2); and Pierot L, 2013, STROKE, V44, P2046 (18).

**Table 3:** The top 10 most cited publications on intracranial aneurysm research from 2012 to 2021.

**Title**	**Author**	**Journal**	**Year**	**Total citations**
The Natural Course of Unruptured Cerebral Aneurysms in a Japanese Cohort	Morita, A.	New England Journal Of Medicine	2012	762
European Stroke Organization Guidelines for the Management of Intracranial Aneurysms and Subarachnoid Haemorrhage	Steiner, T.	Cerebrovascular Diseases	2013	529
Development of the PHASES score for prediction of risk of rupture of intracranial aneurysms: a pooled analysis of six prospective cohort studies	Greving, JP.	Lancet Neurology	2014	524
Endovascular Treatment of Intracranial Aneurysms With Flow Diverters A Meta-Analysis	Brinjikji, W.	Stroke	2013	505
High WSS or Low WSS? Complex Interactions of Hemodynamics with Intracranial Aneurysm Initiation, Growth, and Rupture: Toward a Unifying Hypothesis	Meng, Hui.	American Journal of Neuroradiology	2014	377
Guidelines for the Management of Patients With Unruptured Intracranial Aneurysms A Guideline for Healthcare Professionals From the American Heart Association/American Stroke Association	Thompson, B.G	Stroke	2015	371
Treatment of Intracranial Aneurysms Using the Pipeline Flow-Diverter Embolization Device: A Single-Center Experience with Long-Term Follow-Up Results	Saatci, I.	American Journal of Neuroradiology	2012	302
Biology of intracranial aneurysms: role of inflammation	Chalouhi, N	Journal of Cerebral Blood Flow And Metabolism	2012	288
The durability of endovascular coiling vs. neurosurgical clipping of ruptured cerebral aneurysms: 18 year follow-up of the UK cohort of the International Subarachnoid Aneurysm Trial (ISAT)	Molyneux, A.J.	Lancet	2015	281
Pipeline embolization device (PED) for neurovascular reconstruction: initial experience in the treatment of 101 intracranial aneurysms and dissections	Fischer, S.	Neuroradiology	2012	280

## Discussion

This study employed WoSCC to search for IA-related articles over the recent decade and revealed an upward trend in article quantity between 2012 and 2021. However, the slight decrease in 2020 is noticeable. This is because the COVID-19 pandemic occurred in 2019, which lowered patients' visits to medical facilities due to lockdown and social isolation, and paused clinical and basic research.

The top three countries with high annual production in 2012 are the USA, Japan, and China (19). The growing annual production of China nearly equaled that of the USA in 2021 (199 and 200), which comes down to the following reasons. First, there is a high possibility that more patients are suffering from IA in China because of its largest and most aging population, as well as the high incidence of unruptured IA among Chinese adults aged 35–75 (7%) (2). Second, the number of neurologists and neurosurgeons in China increased sharply in recent years. Data from the World Federation of Neurosurgical Societies (WFNS) showed that China was the largest group of neurosurgeons (*n* = 11,000) in the world since 2016 (20), indicating that more researchers are devoted to the IA field. Third, more government funding and public attention to IA in China were found with the continuous growth in medicine and life quality. The increasing number of papers in China, however, does not necessarily stand for highly influential papers, researchers, and institutions. For example, only one Chinese institution is listed in the top 10 most productive institutions, and two Chinese researchers occupy the top 10 most productive authors. Instead, from a macroscopic viewpoint, China's rank has been raised from the 8th (1991–2012) (19) to the 2nd (2012–2021) in total citations, which indicates significant progress in the last decade.

Unsurprisingly, the USA dominated the field, regardless of publications, total citations, prolific researchers, and institutions. As the world's largest economic entity, the USA is equipped with advanced scientific equipment, professional researchers, and the essential federal funds for scientific research; those factors contributed to its dominant role in the field of IA. The same is true for other fields, such as lumbar spinal stenosis (21) and adolescent idiopathic scoliosis (22). With regards to cooperation between countries, extensive collaborative relationships between the USA, China, Canada, Japan, and some European countries were observed, which validated their significant contributions and probability of producing influential IA-related works in the future.

Leading authors, institutions, and journals provided essential information about a certain field. Table 2 demonstrates the top 10 prolific authors and co-cited authors from 2012 to 2021, who are influential persons leading IA research and accelerating the progress in this field. In addition, the cooperative network of the authors visualized by VOSviewer (Figure 5A) provided information about the productivity and active period of the authors. Several Chinese researchers (e.g., Xinjian, Yang, Jianmin, Liu, Aihua, and Liu) played an active role in the late phase of the recent decade. Such dynamic change in researchers indirectly reflected China's growing position in the field of IA. In terms of journals, eight journals were found in both the top 10 list and the co-cited list, which deserves more attention among researchers due to the influential leading articles that may be published in them. With regard to institutions, Capital Medical University produced the highest number of publications, Mayo Clinic held the top spot in total citations over the recent decade, and other institutions included in the top 10 were engaged in the study of IA and served as potential partners.

A quick overview of a certain field can be realized through keyword analysis and highly cited articles (23). The major topics obtained from VOSviewer author keyword co-occurrence revealed the structure of the IA research knowledge base. Specifically, the three largest clusters almost represented the major topics in this field.

Cluster# 1 (red and green in Figure 6A): *research on the clinical treatment in IA*. Over the past decade, there has been much effort made to identify the best treatment for ruptured and unruptured IA, such as clipping or endovascular treatment (EVT), coiling, or stenting. Technological innovations and advancements in the neuroendovascular space shifted the standard for treating IA from open surgical approaches to endovascular therapies (24). EVT was mentioned in four articles in the top 10 most-cited articles and three co-cited references with the highest citation bursts (ending in 2021). For example, Pierot et al. (18) comprehensively reviewed the status of EVT for IA in 2013. Spetzler et al. (15) reported the 6-year results of the barrow ruptured aneurysm trial (BRAT), proving EVT of coil embolization's superiority to surgery and the higher retreatment rate in the coiling embolization group. Kallmes et al. (16) analyzed the neurological complication rate after pipeline embolization device placement and validated the higher rate of procedure-related morbidity and mortality in posterior circulation IA and giant IA. More importantly, the references with the highest citation bursts over the recent decade also described the detailed outcomes of endovascular coiling vs. clipping, supporting the higher risk of recurrent bleeding and a significantly lower 5-year death rate in the coiling group than in the clipping group (14). Specifically, the green cluster focused mainly on the flow diverter, and the keyword bursts in CiteSpace also showed that flow diversion ended in 2021, suggesting that the flow diverter received huge attention from IA researchers in recent years. In addition, three in five co-cited references published in 2021 discussed the application of flow diverter in IA (25–27). The widespread clinical use of flow diverters also brought more attention to their complications and limitations, such as delayed rupture (28, 29), thromboembolic complications, the need for prolonged antiplatelet therapy (30), and in-stent stenosis (31, 32). Therefore, the application of flow diverter in different types of IA should be expanded in future research (e.g., fusiform aneurysm and dissecting aneurysm), reducing the risk of complications.

Cluster# 2 (purple in Figure 6A): *mechanism research in IA*. The etiopathogenetic features of IA have also attracted much interest, such as inflammation and hemodynamics. Clinically, it is almost unpredictable whether and when rupture will happen as the majority of IAs are silent (3). Furthermore, the progression and rupture of IA were highly correlated with hemodynamic stress and inflammation (33). Therefore, studies of gene expression, hemodynamic characterization, and diagnostic biomarkers were active in recent years. Despite the absence of basic research among the most cited articles, several highly cited reviews should be noted. For instance, Meng et al. (34) reported that different hemodynamics, such as low wall shear stress or high wall shear stress, can crosstalk with different types of inflammable cells or vascular mural cells, mediating the initiation, growth, and rupture of IA. Chalouhi et al. (35) comprehensively discussed the role of inflammation in the biology of IA. Frösen et al. (17) highlighted the crucial role of smooth muscle cells (SMCs) in the formation, degeneration, and rupture of IA. Besides, the authors pointed out that more functioning SMCs or neointimal cells in the wall of the IA can compensate for the constant injury caused by hemodynamic stress and protease activity induced by inflammable cells. Although there is still a long way to go before these findings can be used in clinics, we also hope for more breakthroughs in basic research in the future.

Cluster# 3 (purple in Figure 6A): *complications in IA* (e.g., aneurysmal subarachnoid hemorrhage (aSAH), delayed cerebral ischemia (DCI), and vasospasm) also received considerable attention. Cerebral vasospasm is common and leads to DCI. Hansen-Schwartz et al. reported that DCI should be most blamed for poor outcomes of patients who survive the initial strike of a ruptured IA (36). Thus, extensive studies investigating the biomarkers for DCI in recent 10 years, such as Al-Mufti et al. (37), reported that white cell counts over 12.11 × 0^9^/L were the strongest predictor of DCI. Suzuki et al. (38) reported that higher pH in cerebrospinal fluid (CSF) and lower partial pressure of carbon dioxide (PCO_2_) in CSF acted as a new potential contributor to the development of DCI. Other studies also summarized the role of genetic polymorphisms (39), imaging markers (40), clinical features (41), and serum biomarkers (42–44) in predicting DCI. Such results, however, were primarily obtained by single-center studies, failing to consider two essential attributes of a clinical biomarker, i.e., practicality and robustness. As a result, future prospective studies with multiple centers and larger cohorts should be conducted to validate these clinical biomarkers.

There are several limitations to this study.

The first is the inevitable omittance of publications concerning aSAH or several IA subtypes (e.g., carotid aneurysm, middle cerebral artery aneurysm, basilar aneurysm, anterior communicating aneurysm, etc.) because of the search term “intracranial aneurysm or cerebral aneurysm or brain aneurysm” in the title used to sort relevant articles. The second limitation is the failure to include other widely-accepted databases, such as PubMed, Scopus, and Google Scholar. This is because WoSCC provides the most comprehensive information regarding authors, institutions, and, especially, cited references, which best fit the data format of VOSviewer and CiteSpace. Third, the number of citations and H-index are influenced by time and remain controversial as a comprehensive indicator of the quality of one paper or the author (45). Fourth, the exclusion of articles written in languages other than English resulted in a language bias. Finally, the records update in WoSCC may result in retrieving discrepancies. However, the impact of these new publications was thought to be insignificant on the findings because of their low citations and our huge amount (thousands) of data. It is hypothesized that the bibliometric analysis will provide valuable insights into key points of IA research and future trends for researchers who are already engaged in the field or about to begin.

## Conclusion

In summary, the current research provides a comprehensive analysis of the publications of IA from 2012 to 2021. The main topic of IA research is the choice of EVT or surgical clipping, particularly the application of flow diverter and its complications. More attention should be paid to research on the etiopathogenesis of IA from the perspective of inflammation-related Genes and hemodynamics.

## Data availability statement

The original contributions presented in the study are included in the article/Supplementary material, further inquiries can be directed to the corresponding author/s.

## Author contributions

QZ: conceptualization, methodology, funding acquisition, data curation, writing original draft, study findings, and read and approved the final version before submission. LW: data curation, writing review and editing, study findings, and read and approved the final version before submission. JL: conceptualization, methodology, supervision, critical review of the manuscript, study findings, and read and approved the final version before submission. All authors contributed to the article and approved the submitted version.

## Funding

This research was supported by National Natural Science Foundation of China (No. 81902551) and Natural Science Foundation of Hunan Province (No. 2021JJ31072).

## Conflict of interest

The authors declare that the research was conducted in the absence of any commercial or financial relationships that could be construed as a potential conflict of interest.

## Publisher's note

All claims expressed in this article are solely those of the authors and do not necessarily represent those of their affiliated organizations, or those of the publisher, the editors and the reviewers. Any product that may be evaluated in this article, or claim that may be made by its manufacturer, is not guaranteed or endorsed by the publisher.
