# Supplementary material for: The evolution of intracranial aneurysm research from 2012 to 2021: Global productivity and publication trends
Source: Front Neurol. 2022 Sep 28;13:953285. doi: 10.3389/fneur.2022.953285 (PMC9554263; doi:10.3389/fneur.2022.953285)
Supplement: Supplementary file 1 [file Table_1.docx]

Table S1. Thesaurus

| label | replace by |  |
| --- | --- | --- |
| intracranial aneurysms | intracranial aneurysm |  |
| cerebral aneurysms | intracranial aneurysm |  |
| cerebral-artery aneurysms | intracranial aneurysm |  |
| cerebral aneurysm | intracranial aneurysm |  |
| brain aneurysm | intracranial aneurysm |  |
| induced cerebral aneurysms | intracranial aneurysm |  |
| unruptured cerebral aneurysms | unruptured intracranial aneurysm | |
| aneurysm | intracranial aneurysm |  |
| rabbit model | rabbit |  |
| rabbits | rabbit |  |
| abdominal aortic-aneurysms | abdominal aortic-aneurysm |  |
| macrophages | macrophage |  |
| rats | rat |  |
| mouse model | mice |  |
| shear-stress | wall shear stress |  |
| wall shear-stress | wall shear stress |  |
| hemodynamic-changes | hemodynamics |  |
| hemodynamic | hemodynamics |  |
| kappa-b | nf-kappa-b |  |
| factor-kappa-b | nf-kappa-b |  |
| inflammatory response | inflammation |  |
| cfd | computational fluid dynamics |  |
| computational fluid-dynamics | computational fluid dynamics | |
| mra | MRA | |
| magnetic resonance imaging | MRI |  |
| magnetic resonance-angiography | MRA |  |
| magnetic-resonance angiography | MRA | |
| ct angiography | CTA | |
| computed tomography angiograph | CTA |  |
| multiple intracranial aneurysms | multiple aneurysms |  |
| bypass | bypass surgery |  |
| extracranial-intracranial bypass | bypass surgery |  |
| giant intracranial aneurysm | giant aneurysm |  |
| recurrences | recurrence |  |
| stents | stent |  |
| stenting | stent |  |
| risk factor | risk factors |  |
| risk-factors | risk factors |  |
| flow diversion | flow diverter | |
| diversion | flow diverter |  |
| complications | complication |  |
| subarachnoid haemorrhage | subarachnoid hemorrhage |  |
